# H_2_O_2_ Sensitivity of K_v_ Channels in Hypoxic Pulmonary Vasoconstriction: Experimental Conditions Matter

**DOI:** 10.3390/ijms26146857

**Published:** 2025-07-17

**Authors:** Ornella Tchokondu Yamdjeu, Anouk Begerow, Natascha Sommer, Martin Diener, Norbert Weissmann, Fenja Knoepp

**Affiliations:** 1 Excellence Cluster Cardio-Pulmonary Institute, Universities of Giessen and Marburg Lung Center, Member of the German Center for Lung Research, Justus Liebig University Giessen, 35392 Giessen, Germany; 2 Institute for Veterinary Physiology and Biochemistry, Justus Liebig University Giessen, 35392 Giessen, Germany

**Keywords:** hypoxic pulmonary vasoconstriction (HPV), K_v_ channels, K_v_β subunits, hydrogen peroxide (H_2_O_2_), two-electrode voltage clamp (TEVC), *Xenopus laevis* oocytes

## Abstract

Hypoxic pulmonary vasoconstriction (HPV) optimizes gas exchange but, when impaired, can result in life-threatening hypoxemia. Moreover, under conditions of generalized alveolar hypoxia, HPV can result in pulmonary hypertension. Voltage-gated K^+^ channels (K_v_ channels) are key to HPV: a change in the intracellular hydrogen peroxide (H_2_O_2_) levels during acute hypoxia is assumed to modulate these channels’ activity to trigger HPV. However, there are longstanding conflicting findings on whether H_2_O_2_ inhibits or activates K_v_ channels. Therefore, we hypothesized that H_2_O_2_ affects K_v_ channels depending on the experimental conditions, i.e., the H_2_O_2_ concentration, the channel’s subunit configuration or the experimental clamping potential in electrophysiological recordings. Therefore, cRNAs encoding the K_v_1.5 channel and the auxiliary K_v_β subunits (K_v_β1.1, K_v_β1.4) were generated via in vitro transcription before being injected into *Xenopus laevis* oocytes for heterologous expression. The K^+^ currents of homomeric (Kv1.5) or heteromeric (K_v_1.5/K_v_β1.1 or K_v_1.5/K_v_β1.4) channels were assessed by two-electrode voltage clamp. The response of the K_v_ channels to H_2_O_2_ was markedly dependent on (a) the clamping potential, (b) the H_2_O_2_ concentration, and (c) the K_v_ channel’s subunit composition. In conclusion, our data highlight the importance of the choice of experimental conditions when assessing the H_2_O_2_ sensitivity of K_v_ channels in the context of HPV, thus providing an explanation for the long-lasting controversial findings reported in the literature.

## 1. Introduction

Hypoxic pulmonary vasoconstriction (HPV) is a physiological mechanism of the mammalian lung that optimizes gas exchange. By rapidly matching perfusion to ventilation, HPV ensures optimal oxygenation of the blood during local alveolar O_2_ shortage, i.e., hypoxia [1,2,3]. Impairment of this vital mechanism can result in insufficient blood oxygenation and thus O_2_ undersupply to the organs. Such fatal hypoxemia due to disturbed HPV can occur in various clinical scenarios. This includes patients afflicted with pneumonia, those with acute respiratory distress syndrome (ARDS), or those requiring mechanical ventilation, e.g., COVID-19 patients during the recent pandemic [4]. Additionally, systemic administration of pulmonary vasodilatory agents, such as prostacyclin, has been associated with similar adverse outcomes due to impaired HPV [2,5]. Moreover, imbalanced HPV is considered to provoke high-altitude pulmonary edema (HAPE), a life-threatening condition [5,6,7], and also acquires pathophysiological significance in situations with prolonged hypoxia, e.g., at high altitudes or in lung diseases such as sleep apnea or chronic obstructive pulmonary disease (COPD) [8,9,10]. In these conditions, generalized and consistent alveolar hypoxia can occur, leading to the constriction of the blood vessels in the entire lung. If hypoxia persists, the subsequent onset of vascular remodeling processes leads—in addition to vasoconstriction—to the manifestation of pulmonary hypertension, which can ultimately result in right heart failure.

Although HPV was already described in 1946 by von Euler and Liljestrand, the underlying molecular mechanisms are not yet fully known [3,11]. A mandatory step in HPV is the hypoxia-induced inhibition of O_2_-sensitive voltage-gated potassium (K_v_) channels in pulmonary artery smooth muscle cells (PASMCs) [12,13,14,15,16]. K_v_ channels are composed of four α subunits forming a central pore in the membrane and four auxiliary β subunits to fine-tune the channel’s activity [13,17]. By mediating constant K^+^ leakage, K_v_ channels significantly contribute to maintaining the cellular membrane potential [16,18,19]. It is undisputed that hypoxia induces the closure of K_v_ channels, which in turn depolarizes the membrane potential of PASMCs, thereby increasing the Ca^2+^ influx via the activation of voltage-gated Ca^2+^ channels [2,20,21]. The resulting increase in intracellular Ca^2+^ induces the constriction of the individual cells and thus the pulmonary arterial vessels. Although much progress has been made in understanding the signaling pathways underlying O_2_ sensing in HPV, the molecular mechanisms by which hypoxia affects the K_v_ channel activity are still not fully resolved [13].

A proposed signaling mechanism linking hypoxia to the K_v_ channel function in PASMCs involves reactive oxygen species (ROS) and hydrogen peroxide (H_2_O_2_) in particular [3,13,22,23,24]. Although there is a consensus on the involvement of ROS in hypoxia-induced K_v_ channel inhibition, there is a long-lasting disagreement as to whether hypoxia induces an increase or decrease in ROS [2,13]. The original redox hypothesis of Archer, Weir, and colleagues proposes that under normoxic conditions, a certain level of intracellular ROS, i.e., H_2_O_2_, maintains the K_v_ channels in an oxidized open state. Conversely, acute hypoxia causes an increase in the NADH/NAD^+^ ratio and a concomitant decrease in the H_2_O_2_ level that shifts the cellular redox state to a more reduced state, resulting in K_v_ channel inhibition and subsequent vasoconstriction [12,17,25,26,27]. In contrast, Paul Schumacker’s lab presented an opposing concept, often referred to as the mitochondrial ROS theory, which describes a hypoxia-induced increase in the intracellular ROS levels being causative for the inhibition of K_v_ channels [13,28]. This theory was not only confirmed by previous studies by our research group, but we could also identify the signaling molecule responsible as H_2_O_2_ [3] and further decipher the role of mitochondria in this regard [3,22]. Although there is a large body of evidence for increased ROS levels during acute hypoxia underlying HPV, both theories emphasize that the K_v_ channels are inhibited by the indisputable hypoxia-induced change in the intracellular H_2_O_2_ levels [3,13].

Consequently, several studies investigated the influence of H_2_O_2_ on K_v_ channel activity. In line with the discrepancies between the two distinct theories, contrary results were also reported by distinct research groups in this context. While some studies showed an activation of the K_v_ channels in response to H_2_O_2_ [29,30,31], other groups observed an H_2_O_2_-induced inhibition of these channels [3,32,33,34]. Upon thorough examination of these reports, discrepancies in the experimental conditions became apparent, specifically the widely varying and occasionally nonphysiologically high concentrations of H_2_O_2_ utilized. Furthermore, the studies were conducted at distinct experimental clamping potentials and on very different cell types, including various primary cells as well as overexpression systems that may be featured by different subunit compositions of K_v_ channels. In particular, the auxiliary K_v_β subunits are differentially expressed in the pulmonary circulation [35]. The reported correlation between their expression level and the strength of HPV suggests a role for these subunits in O_2_ sensitivity [35]. Thus, the presence or absence of K_v_β subunits could be responsible for the different responses of K_v_ channels to H_2_O_2_, thereby delivering a potential explanation for the contrary results described in the literature.

Considering the controversy as to whether H_2_O_2_ inhibits or activates K_v_ channels, we hypothesized that H_2_O_2_ regulates K_v_ channel activity (a) in a concentration-dependent manner and/or (b) in a manner dependent on the molecular composition of the channel, i.e., the presence or absence of auxiliary K_v_β subunits.

## 2. Results

### 2.1. Homomeric Kvβ Subunits Do Not Form Functional Kv Channels

**Figure 1:**
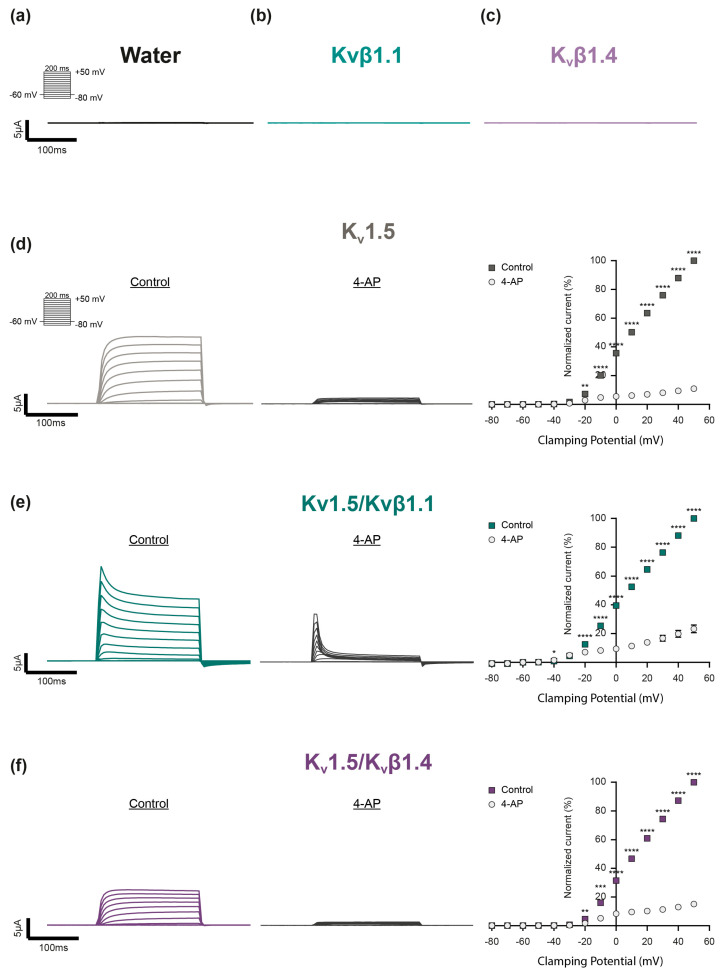
Expression and electrophysiological characterization of distinct K_v_ channel combinations in *Xenopus laevis* oocytes. (**a**–**c**) Representative current traces of control oocytes exposed to depolarizing voltage steps. Neither (**a**) water (black) nor (**b**) homomeric K_v_β1.1 (light turquois) or (**c**) K_v_β1.4 subunits (light purple) built functional channels. (**d**–**f**) Electrophysiological characterization of (**d**) homomeric K_v_1.5 channels (gray), (**e**) K_v_1.5 co-expressed with the auxiliary subunits K_v_β1.1 (K_v_1.5/K_v_β1.1, dark turquoise) or (**f**) together with K_v_β1.4 (K_v_1.5/K_v_β1.4, dark purple). Current traces were elicited by depolarizing voltage pulses (200 ms) between −80 mV and +50 mV in 10 mV steps from a holding potential of −60 mV before (Control) and after application of the K_v_ channel inhibitor 4-aminopyridine (4-AP) to validate the successful K_v_ channel expression. The current–voltage relationship (I–V curve) was determined by plotting the averaged current amplitude (plateau) against the corresponding voltage step (K_v_1.5: n = 13; K_v_1.5/K_v_β1.1: n = 12; K_v_1.5/K_v_β1.4: n = 13). The mean K_v_ current amplitudes were each normalized relatively to the current value at + 50 mV under control conditions (no 4-AP). Data were statistically analyzed by 2-way ANOVA and the uncorrected Fisher’s least significant difference test for multiple comparisons (*: *p* ≤ 0.05; **: *p* ≤ 0.01, ***: *p* ≤ 0.001; ****: *p* ≤ 0.0001).

To address these questions, we selected the K_v_1.5 channel as a suitable candidate. K_v_1.5 has long been recognized as a channel involved in HPV [36,37]. In line with this, mice lacking K_v_1.5 exhibited impaired HPV [38], which could be fully restored by its subsequent introduction via gene transfer in vivo [39]. To address the H_2_O_2_ sensitivity of K_v_1.5, this subunit was heterologously expressed either as a homomeric or heteromeric channel, i.e., in combination with either K_v_β1.1 or K_v_β1.4 subunits in *Xenopus laevis* oocytes, which are supposed to not express endogenous K_v_ channels [40,41]. The potassium (K^+^) currents were assessed via the two-electrode voltage clamp technique (TEVC) by applying depolarizing 200 ms voltage pulses from a holding potential of −60 mV to +50 mV in 10 mV steps. Neither water-injected control oocytes (Figure 1a) nor oocytes injected with the auxiliary subunits K_v_β1.1 (Figure 1b) or K_v_β1.4 (Figure 1c) alone exhibited voltage-dependent currents when depolarized. In contrast, oocytes injected with cRNA-encoding K_v_1.5 subunits showed typical rapid outward K^+^ currents upon depolarization to potentials more positive than −20 mV (Figure 1d). In agreement with previous studies, the homomeric K_v_1.5 currents exhibited no detectable inactivation in response to such short pressure pulses [42]. Co-expression with K_v_β1.1 altered the K_v_1.5 activity by inducing a rapid voltage-dependent inactivation (Figure 1e), while co-expression with K_v_β1.4 lowered the currents’ amplitude (Figure 1f). The final application of 4-aminopyridine (4-AP) largely inhibited the K^+^ currents (Figure 1d–f), which confirmed the successful expression of the three K_v_ channel combinations.

### 2.2. Evaluation of H_2_O_2_ Decomposition in the Experimental Setup

**Figure 2:**
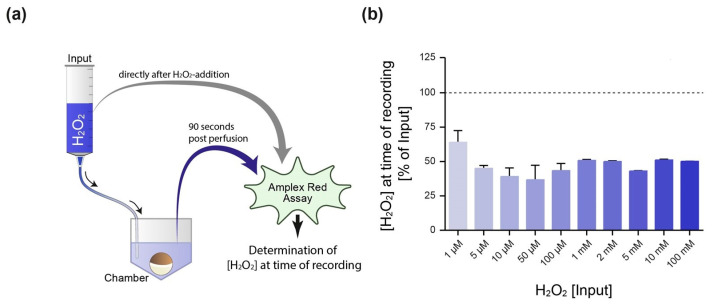
Determination of the H_2_O_2_ concentration present at the time point of electrophysiological recording. (**a**) Schematical illustration of the experimental setup. Oocyte Ringer’s solution (ORi) was supplemented with H_2_O_2_ within the perfusion reservoir (input). One sample was taken directly after H_2_O_2_ addition (input), while the second sample was extracted from the recording chamber 90 s post starting the perfusion—which represents the time point at which the electrophysiological recording was performed. Both samples were subjected to an Amplex Red Hydrogen Peroxide/Peroxidase Assay to determine the H_2_O_2_ decomposition for distinct concentrations within the experimental setup, i.e., the H_2_O_2_ concentration at the time of recording (**b**). The dotted line represents the input concentration (100%).

Beside the temperature and the presence of decomposing impurities, the stability of H_2_O_2_ in aqueous solution is affected by the pH value [43]. For optimum stability, the pH of pure H_2_O_2_ is below pH 4.5, while increasing it to above pH 5 sharply increases the decomposition of H_2_O_2_ into water and oxygen. Since the pH value of the oocyte Ringer’s solution (ORi) used in our experiments was at a physiological value of pH 7.4, we first tested the decomposition of H_2_O_2_ in our experimental setup via an Amplex Red Hydrogen Peroxide/Peroxidase Assay. As schematically shown in Figure 2a, the ORi in the reservoir was analyzed directly after adding H_2_O_2_ to the perfusate (input). Afterwards, the perfusion was started to deliver the H_2_O_2_-containing ORi to the recording chamber. The second sample was then taken from the recording chamber 90 s post starting the perfusion—which corresponds to the time point at which the K^+^ currents were recorded in the electrophysiological experiments. We observed that at the time point of current recording, approximately 50% of the H_2_O_2_ of the distinct input concentrations was decomposed (Figure 2b).

### 2.3. Experimental Approach to Evaluate the Effect of H_2_O_2_ on K_v_ Channel Activity

**Figure 3:**
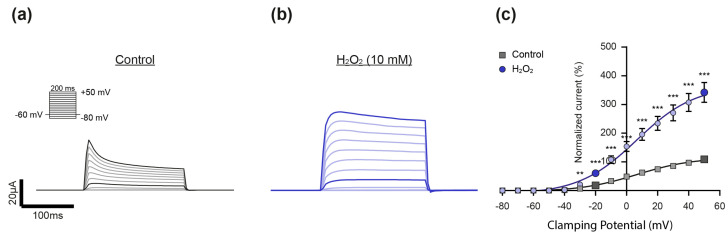
Experimental approach to evaluate the effect of H_2_O_2_ on K_v_ channel activity. (**a**) Representative current trace of a K_v_-channel-expressing oocyte that was exposed to depolarizing voltage steps in the (**a**) absence (Control, gray) and (**b**) presence of H_2_O_2_ (10 mM, blue), as well as (**c**) the corresponding statistical analysis. The current–voltage relationship (I–V curve) was determined by plotting the averaged current amplitude (plateau) against the corresponding voltage step (control: n = 12; H_2_O_2_: n = 11). The I–V curve was fitted using the Boltzmann equation. No differences in the V_50_ (Control: 7.1 ± 1.6 mV, H_2_O_2_; 6.1 ± 1.4 mV; *p* = 0.6523) and gating charge (q) (control: 1.4 ± 0.038 e_0_, H_2_O_2_: 1.4 ± 0.028 e_0_; *p* = 0.4252) were observed in response to H_2_O_2_. The mean K_v_ current amplitudes were each normalized relatively to the current amplitude in the control condition at +50 mV. H_2_O_2_ increased the channel’s activity at clamping potentials more positive than −30 mV, with a prominent effect at −20 mV, a value within the physiological range of the membrane potential of PASMCs. Therefore, clamping potentials of −20 mV and +50 mV (characterized by the strongest H_2_O_2_-induced activation) were chosen as the clamping potentials for analysis in all the following experiments (respective current traces and data points are highlighted in darker colors). Data were statistically analyzed by 2-way ANOVA and uncorrected Fisher’s least significant difference test for multiple comparisons (**: *p* ≤ 0.01, ***: *p* ≤ 0.001).

For evaluation of the effect of H_2_O_2_ on K_v_ channel activity, K_v_ currents were elicited by depolarizing voltage pulses between −80 mV and +50 mV in 10 mV steps from a holding potential of −60 mV in the absence (Control, Figure 3a) and after the addition of H_2_O_2_ (Figure 3b). As shown in Figure 3c, H_2_O_2_ (10 mM) significantly activated K^+^ currents at all the clamping potentials of −30 mV and more positive—with a prominent effect at −20 mV, a value within the physiological range of the membrane potential of PASMCs under hypoxia [3,23]. Therefore, two distinct voltage steps were chosen for statistical analysis in all the following experiments: the one that lies within the physiological range of the membrane potential of PASMCs (−20 mV) and one at a positive membrane potential, which was characterized by the strongest H_2_O_2_-induced activation (+50 mV).

### 2.4. The Effect of H_2_O_2_ on Homomeric K_v_1.5 Channels Is Voltage- and Concentration-Dependent

**Figure 4:**
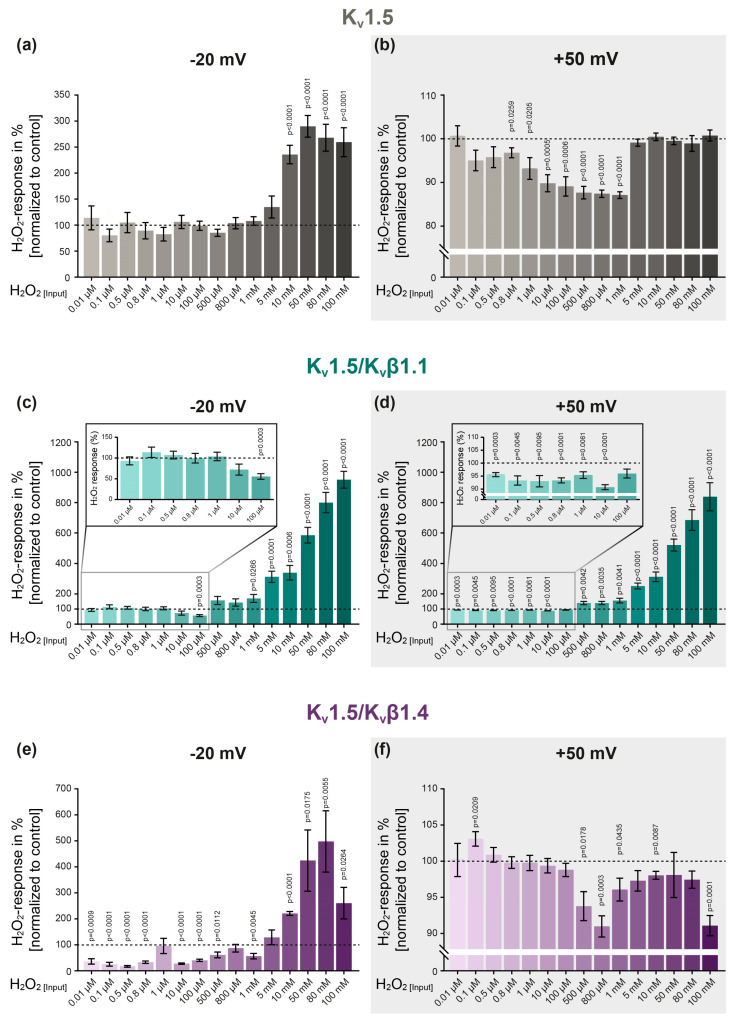
The effect of H_2_O_2_ on K_v_ channels is dependent on the concentration, step potential and ion channel subunit composition. (**a**) The effect of increasing H_2_O_2_ concentrations on homomeric K_v_1.5 channels at step potentials of −20 mV and (**b**) +50mV (light gray background). (**c**,**d**) Assessment of the H_2_O_2_ response of heteromeric K_v_1.5/K_v_β1.1 as well as (**e**,**f**) K_v_1.5/K_v_β1.4 channels. The H_2_O_2_ response was assessed by normalizing the current amplitude upon H_2_O_2_ application to the control conditions (represented by the dotted line). A new oocyte and freshly prepared H_2_O_2_ were used for each experiment. Number of experiments per H_2_O_2_ concentration: n = 8–13 (K_v_1.5); n = 8–13 (K_v_1.5/K_v_β1.1); n = 6–13 (K_v_1.5/K_v_β1.4). For statistical analysis, one-sample *t*-tests were performed, with *p* ≤ 0.05 being considered indicative of significance. Group data are expressed as the mean ± SEM.

To assess the effect of distinct H_2_O_2_ concentrations on the activity of homomeric K_v_1.5 channels, H_2_O_2_ was applied in concentrations between 0.01 µM and 100 mM (Figure 4a,b), using a new oocyte and freshly prepared H_2_O_2_ for each individual experiment. As previously described, the K^+^ current amplitudes were analyzed at step potentials of −20 mV (Figure 4a) and +50 mV (Figure 4b) to determine the potential voltage-dependency of the H_2_O_2_ effect. In our investigation, we observed dependencies on both voltage and concentration: while low H_2_O_2_ concentrations (ranging from 0.1 µM to 1 mM) did not affect the activity of K_v_1.5 channels at a physiological step potential of −20 mV (Figure 4a), these same concentrations were found to inhibit the channels at a positive step potential (+50 mV, Figure 4b). Interestingly, higher H_2_O_2_ concentrations (10 mM and above) were observed to enhance the K^+^ currents at −20 mV (Figure 4a), yet no such effect was noted for the same concentrations at +50 mV (Figure 4b). These findings underscore a dual relationship involving both the voltage- and concentration-dependency of H_2_O_2_ modulation on K_v_1.5 channel activity.

### 2.5. Co-Expression of K_v_1.5 with K_v_β Subunits Modulates the Channel’s Response to H_2_O_2_

To determine whether co-expression with accessory β subunits alters the channel’s response to H_2_O_2_, K_v_1.5 was co-expressed with the β-isoform K_v_β1.1 and the channels’ response to H_2_O_2_ was assessed by TEVC (Figure 4c,d). While low H_2_O_2_ concentrations (0.01 µM–10 µM) did not affect the activity of the K_v_1.5/K_v_β1.1 channels at a step potential of −20 mV (Figure 4c), the same concentrations significantly inhibited the K_v_1.5/K_v_β1.1 currents at +50 mV (see enlargement in Figure 4d). Importantly, this inhibition was induced even with the lowest H_2_O_2_ concentration, i.e., 0.01 µM. In contrast, application of higher H_2_O_2_ concentrations (1 mM to 100 mM) significantly and concentration-dependently increased the K^+^ currents at both step potentials, i.e., −20 mV and +50 mV (Figure 4c,d).

To assess whether distinct isoforms differently modulate the response of K_v_1.5 to H_2_O_2_, an additional K_v_β isoform (K_v_β1.4) was co-expressed with K_v_1.5 (Figure 4e,f). In contrast to the homomeric K_v_1.5 and heteromeric K_v_1.5/K_v_β1.1 channels, this channel combination was significantly inhibited by low H_2_O_2_ concentrations at −20 mV (Figure 4e). Importantly, this inhibition occurred under physiologically relevant conditions in terms of both the clamping potential and the H_2_O_2_ concentration. Notably, K_v_1.5/K_v_β1.4 was the only channel combination that remained unaffected by low H_2_O_2_ concentrations at a depolarized clamping potential (+50 mV) while being inhibited when exposed to high concentrations of 500 µM and above (Figure 4f).

## 3. Discussion

It is well accepted that a shift in the intracellular ROS levels in response to acute hypoxia inhibits K_v_ channels in PASMCs, thereby triggering HPV. However, although the signaling molecule responsible could be identified as H_2_O_2_ [3], long-lasting divergences and contradictory reports exist in the literature on whether H_2_O_2_ inhibits or activates K_v_ channels. We hypothesized that these contrary observations depend on (a) the experimental clamping potential, (b) the H_2_O_2_ concentration utilized and/or (c) the K_v_ channel subunit composition, i.e., the presence of auxiliary K_v_β subunits. Therefore, the aim of the present study was to determine whether H_2_O_2_ modulates K_v_ channel activity in a concentration-dependent manner, and at the same time, to address the potential role of the accessory β subunits K_v_β1.1 and K_v_β1.4 as well as the experimental clamping potential in H_2_O_2_ sensitivity. For our studies, we heterologously expressed K_v_1.5 as a homomeric channel and in combination with either K_v_β1.1 or K_v_β1.4, respectively, before exposing them to H_2_O_2_ at various concentrations between 0.01 µM and 100 mM. We then analyzed the K_v_ currents at two distinct clamping potentials, i.e., (a) at −20 mV, which falls within the physiological resting membrane potential of PASMCs and (b) at +50 mV, which was characterized by the highest voltage-induced current.

As an expression system, we used *Xenopus laevis* oocytes that are supposed to not express endogenous 4-AP-sensitive K_v_ channels [40,41]. This assumption was confirmed in our study as neither the water-injected control oocytes, nor the oocytes injected with K_v_β subunits alone exhibited voltage-induced currents upon depolarization, thereby confirming the choice of the expression system as ideally suited for investigating the effect of H_2_O_2_ on defined K_v_ channel combinations.

### 3.1. The Reaction of K_v_ Channels to H_2_O_2_ Depends on the Experimental Clamping Potential

K_v_ currents are typically measured at positive clamping potentials of up to +70 mV [29,34], which not only ensures channel activation but also maximizes the driving force for K^+^ efflux, thereby facilitating current detection and analysis. However, such positive clamping potentials do not reflect physiological conditions, as the membrane potentials in PASMCs typically range from approximately −50 mV to −10 mV [3,44]. As a result, the K_v_ channel behavior observed at such strongly positive (depolarized) voltages may not fully reflect their natural activation, inactivation, or response to pharmacological modulation under physiological conditions. In line with this, we observed a clear voltage-dependency in the response to H_2_O_2_ for all three channel combinations, i.e., K_v_1.5, K_v_1.5/K_v_β1.1 and K_v_1.5/K_v_β1.4 (see Figure 4). For example, at a depolarizing voltage step of +50 mV, the homomeric K_v_1.5 channels were inhibited by low concentrations of H_2_O_2_ (0.8 µM–1 mM), while higher H_2_O_2_ concentrations (5 mM–100 mM) had no effect on the channels’ activity. In contrast, at a physiological clamping potential of −20 mV, the channels exhibited a completely different response pattern, being unaffected by low H_2_O_2_ concentrations but strongly activated by H_2_O_2_ concentrations of 10 mM and above. Similar observations of voltage-dependent effects were also made for the heteromeric channels, i.e., K_v_1.5/K_v_β1.1 and K_v_1.5/K_v_β1.4. These data clearly indicate that the response of the K_v_ channels to H_2_O_2_ is markedly dependent on the experimental clamping potential.

However, it is important to consider that—in contrast to *Xenopus laevis* oocytes—in both primary cells and overexpression systems (particularly mammalian cell lines), endogenous ion channels might be active at physiological clamping potentials and contribute to the recorded currents, thus potentially confounding the detection and analysis of K_v_-channel-specific responses. Furthermore, at physiological clamping potentials, the electrochemical driving force for K^+^ is diminished, resulting in reduced current amplitudes and less pronounced differences, thereby potentially masking subtle differences that are more apparent under depolarized conditions. Thus, the choice of clamping protocol constitutes a critical experimental parameter that can profoundly affect the biophysical properties and pharmacological response of K_v_ channels to H_2_O_2_.

### 3.2. The Effect of H_2_O_2_ on K_v_ Channel Activity Is Concentration-Dependent

The estimation of the intracellular concentration of H_2_O_2_ is based on the kinetics of production and elimination, while its determination is still technically difficult [45,46]. The physiological estimate of the cytosolic H_2_O_2_ concentration has been reported to be in the pico- to nanomolar range, e.g., 2.2 nM [47], 1–700 nM [48], 1–10 nM [46] or even in the picomolar range [47,49]. Consequently, H_2_O_2_ concentrations above the nanomolar range are regarded as pathological levels that are related to oxidative stress [46].

In previous studies investigating the effect of H_2_O_2_ on K_v_ channel activity, various concentrations of H_2_O_2_ were applied, resulting in contradictory outcomes. Higher concentrations, ranging from 0.1 to 10 mM, were reported to activate K_v_ channels [29,30,31]. In contrast, other studies employing lower concentrations, i.e., between 5 µM and 400 µM, observed an inhibitory effect on K_v_ channel activity [32,33,34]. These divergent observations prompted us to hypothesize that the concentration of H_2_O_2_ used under experimental conditions underlies the reported inconsistencies in H_2_O_2_-mediated K_v_ channel modulation, i.e., whether H_2_O_2_ inhibits or activates K_v_ channels. Based on this rationale, we applied a broad range of H_2_O_2_ concentrations, spanning from physiological to pathophysiological levels (10 nM to 100 mM).

At a physiological clamping potential of −20 mV, we observed the clear concentration-dependency of H_2_O_2_ on the homomeric K_v_1.5 channel activity: whereas H_2_O_2_ did not affect the K_v_1.5 activity at physiological concentrations, an activation of up to 200% was observed in response to pathophysiological concentrations of 10 mM and above. As discussed in the previous paragraph, a different response pattern was observed at a depolarized clamping potential (+50 mV), although there was still a clear difference between the physiological and pathophysiological H_2_O_2_ concentrations. Interestingly, the channel’s response to high doses of H_2_O_2_ appeared to be an “all or nothing” effect as no difference was observed between the concentrations applied. This indicates that oxidation by H_2_O_2_ above a certain level might induce a conformational change within the channel structure that increases the channel’s activity. However, although K_v_1.5 has been implicated in HPV and is therefore presumed to be inhibited by hypoxia-induced H_2_O_2_ release, we did not observe an inhibition of homomeric K_v_1.5 in response to H_2_O_2_ at physiological clamping potentials—regardless of the H_2_O_2_ concentration applied. Given that the accessory β subunits are reported to exert regulatory functions on K_v_ channels, including modulation of their redox sensitivity [50], we next investigated their influence on the H_2_O_2_ sensitivity of K_v_1.5 channels.

### 3.3. K_v_β Subunits Modulate the Response of K_v_1.5 to H_2_O_2_

Interestingly, the H_2_O_2_-induced activation in response to pathophysiological concentrations above 10 mM could be further potentiated by the co-expression of K_v_1.5 with K_v_β1.1. In these heteromeric channels (K_v_1.5/K_v_β1.1), H_2_O_2_ of 1 mM and above induced a channel activation of up to 900%, which in contrast to the homomeric K_v_1.5 channels, was clearly concentration-dependent. This observation suggests that the accessory β subunits not only alter the channel’s activity per se, as described in several studies [51,52,53], but also equip them with the ability to fine-tune the kinetics of K_v_1.5 channels in response to H_2_O_2_, most likely as a result of their oxidoreductase properties [50]. However, an H_2_O_2_-induced inhibition of the channel, as would be necessary for HPV initiation, was only observed in response to an H_2_O_2_ concentration of 100 µM—which, however, is not in the assumed physiological range of intracellular H_2_O_2_ concentrations [46].

The co-expression of K_v_1.5 with the auxiliary isoform K_v_β1.4 rendered the channel sensitive to inhibition by physiological H_2_O_2_ concentrations—even as low as 10 nM. Most interestingly, this inhibition was observed at a physiological clamping potential—thereby closely reflecting the physiological conditions present in PASMCs under hypoxia. Given that H_2_O_2_ was decomposed by approximately 50% under our experimental conditions, this suggests that K_v_1.5/K_v_β1.4 channels can be inhibited by H_2_O_2_ concentrations of less than 10 nM. Sommer et al. reported in a study from our laboratory that 124 nM H_2_O_2_ inhibits the K_v_ currents in primary mouse PASMCs [3], thereby contributing to the initiation of HPV. This finding is consistent with the present data, which demonstrate that even lower concentrations of H_2_O_2_ can significantly inhibit K_v_ currents, an effect that is—at least for Kv1.5—critically dependent on the clamping potential and the ion channel composition, particularly the presence of the auxiliary subunit K_v_β1.4.

It should be noted, however, that in addition to K_v_1.5, a variety of other K_v_ channel subunits contribute to HPV initiation and further studies would be required to investigate the effect of H_2_O_2_ on these channels as well. However, our study clearly highlights the importance of carefully selecting experimental parameters—particularly the necessity of utilizing physiological conditions—when attempting to elucidate a physiological process.

### 3.4. Conclusions

In conclusion, our findings indicate that the response of K_v_ channels to H_2_O_2_ is markedly dependent on (a) the experimental clamping potential, (b) the H_2_O_2_ concentration and (c) the K_v_ channel subunit composition. Thus, our data clearly highlight the importance of the choice of experimental conditions when assessing the H_2_O_2_ sensitivity of K_v_ channels in the context of HPV, thus providing an explanation for the long-lasting controversial findings reported in the literature.

## 4. Materials and Methods

### 4.1. Generation of cRNA for Heterologous Expression of K_v_ Channels

Plasmids encoding murine K_v_1.5 (NM_145983.2), K_v_β1.1 (NM_010597.4) or K_v_β1.4 (NM_001403872.1) in a pTNT expression vector (Promega, Madison, WI, USA) were purchased from the Eurofins gene synthesis service (Eurofins, Ebersberg, Germany) and transformed into competent JM109 *E. coli* cells (Promega, Madison, WI, USA) for amplification. Upon subsequent plasmid purification using the GenElute Plasmid Miniprep Kit (Sigma Aldrich, St Louis, MO, USA) according to the manufacturer’s instructions, the purified plasmids were used for cRNA synthesis via in vitro transcription using the mMESSAGE mMACHINE T7 kit (Invitrogen, Waltham, MA, USA). The resulting full-length capped cRNAs were then purified using the MEGAclear transcription cleaning kit (Invitrogen, Waltham, MA, USA). The cRNAs were stored at –80 °C and diluted in nuclease-free water immediately prior to injection.

### 4.2. Preparation, Storage and Injection of Xenopus Laevis Oocytes

*Xenopus laevis* oocytes at stages V–VI were purchased from Ecocyte Bioscience (Dr. Lohmann Diaclean GmbH, Dortmund, Germany) and plated individually in a 96 Conical Bottom MicroWell Plate (Fischer Scientific, Schwerte, Germany) filled with modified Barth’s solution (MBS, in mM: NaCl 88, KCl 1, CaCl_2_ 0.4, Ca(NO_3_)_2_ 0.33, MgSO_4_ 0.8, TRIS-HCl 5, NaHCO_3_ 2.4, pH 7.4, Dr. Lohmann Diaclean GmbH, Dortmund, Germany) that was supplemented with sodium pyruvate (2.5 mM, Sigma Aldrich, St Louis, MO, USA), penicillin (20 µg/mL, Sigma Aldrich, St Louis, MO, USA) and streptomycin (25 µg/mL, Sigma Aldrich, St Louis, MO, USA). The oocytes were stored in a digital mini-incubator (Gilson, Middleton, WI, USA) at 5–8 °C until use.

At 24–48 h prior to the electrophysiological recordings, the oocytes were injected with K_v_1.5: 0.1 ng/oocyte; K_v_β1.1: 1.75 ng/oocyte; K_v_β1.4: 0.1 ng/oocyte in a total injection volume of 10 nL/oocyte (for K_v_1.5 and K_v_1.5/K_v_β1.4, resp.) and 17.5 nL/oocyte (for K_v_1.5/K_v_β1.1) using the Roboinject fully automated injection system (Multichannel Systems, Reutlingen, Germany). Therefore, the injection needle was filled with mineral oil (Sigma Aldrich, St Louis, MO, USA) before being mounted onto the device. Afterwards, the cRNAs that were diluted in nuclease-free water were drawn into the injection needle and injected into the oocytes. The impalement and injection depth for the cRNAs were defined for all the samples as 550 µm and 450 µm, respectively. The injected oocytes were incubated for 24 h at 18 °C for heterologous expression.

### 4.3. Electrophysiological Recordings via Two-Electrode Voltage Clamp (TEVC)

The K_v_ currents of the cRNA injected oocytes were recorded via the two-electrode voltage clamp (TEVC) technique using an automated TEVC system (Roboocyte2, Multichannel Systems, Reutlingen, Germany). Therefore, the glass capillaries of the measure head were filled with 1 M KCl. Only electrodes with tip resistances between 300 and 800 kΩ were used for the experiments. All the electrophysiological recordings were carried out at room temperature (20–22 °C) under continuous perfusion with oocyte Ringer’s solution (ORi; Normal Frog Ringer, Ecocyte Biosciences, composition in mM: NaCl 64, KCl 2, CaCl_2_ 2, MgCl_2_ 1, NaHCO_3_ 26). The ORi inside the perfusion reservoir was continuously gassed with 21% O_2_, 5.3% CO_2_, 73.7 N_2_ for pH stabilization at pH 7.4. Due to the rapid decomposition of H_2_O_2_ at physiological pH values, the H_2_O_2_ (Sigma Aldrich, St Louis, MO, USA) was freshly prepared directly before the start of each individual experiment. Being a strong oxidizing agent, H_2_O_2_ has the potential to corrode Ag/AgCl electrodes upon direct contact, which would manifest in a shift of the baseline current. Anticipating this potential issue, a rigorous maintenance protocol involving regular and frequent re-chlorination and replacement of the electrodes was implemented. Additionally, the baseline current was continuously monitored to ensure stability throughout the experiments.

For the electrophysiological recordings, the oocytes were voltage-clamped at a holding potential of −60 mV. The current amplitudes were elicited by depolarizing voltage pulses in 10 mV increment steps starting from −80 mV to +50 mV and 200 ms duration.

### 4.4. Experimental Design and Analysis

As a current rundown was already observed in the control recordings (two recordings in succession with an interval of 90 s between the two recordings), a different procedure was used. Two sets of experiments were performed for each experiment. As a control, two recordings were performed in the absence of H_2_O_2_ (control), separated by 90 s. In the second set of experiments, only one control recording was performed, before H_2_O_2_ was delivered to the recording chamber for 90 s. The recording taken after the 90 s (+H_2_O_2_) was then compared with the second current trace from the control measurements (−H_2_O_2_). The recordings +/−H_2_O_2_ were always performed on oocytes obtained from the same individual. The mean K_v_ current amplitudes were normalized relatively to the current at +50 mV in the control experiment. All the current traces were leak-subtracted prior to statistical evaluation. The current–voltage relationship (I–V curve) was obtained by plotting the averaged plateau current against the respective clamping potential. The specific blocker 4-aminopyrimidine (4-AP, 10 mM) was added at the end of experiment to validate the K_v_ channel expression.

### 4.5. Amplex Red Hydrogen Peroxide/Peroxidase Assay

To determine the decomposition of H_2_O_2_ inside the experimental system, H_2_O_2_ was added to the perfusate and delivered to the recording chamber via the perfusion system. At 90 s post starting the perfusion, a sample was taken from the recording chamber, representing the time point where the electrophysiological recording (+H_2_O_2_) was performed. The H_2_O_2_ concentration (input, i.e., [H_2_O_2_] present at time of recording) was determined by Amplex^®^ Red Hydrogen Peroxide/Peroxidase Assay Kit (Thermo Fisher Scientific, Waltham, MA, USA). The assay was performed according to the supplier’s instructions. The absorbance was measured at 560/590 nm using a microplate reader TECAN Spark 10M (Tecan Trading AG, Männedorf, Switzerland). The manuscript consistently reports the input H_2_O_2_ concentration. However, it should be considered that—due to the decomposition of H_2_O_2_—the effective concentration at the oocyte during the electrophysiological recordings is lower (see Figure 2).

### 4.6. Statistical Analysis

The statistical differences were assessed via two-way ANOVA and the uncorrected Fisher’s least significant difference test for multiple comparisons or one-sample *t*-tests. All the analyses were considered statistically significant at *p* ≤ 0.05. Data are expressed as the mean ± SEM. The statistical analysis was performed using Prism 10 (GraphPad Software Inc., San Diego, CA, USA).

## Data Availability

The raw data supporting the conclusions of this article will be made available by the authors on reasonable request.

## Abbreviations

The following abbreviations are used in this manuscript:

ARDS	acute respiratory distress syndrome
cRNA	complementary ribonucleic acid
HPV	hypoxic pulmonary vasoconstriction
I–V curve	current–voltage relationship
K_v_ channel	voltage-gated potassium channel
nM	nanomolar
ORi	oocyte Ringer’s solution
PASMC	pulmonary arterial smooth muscle cell
ROS	reactive oxygen species
TEVC	two-electrode voltage clamp
4-AP	4-aminopyridine
